# Long non-coding RNA LINC00628 functions as a gastric cancer suppressor via long-range modulating the expression of cell cycle related genes

**DOI:** 10.1038/srep27435

**Published:** 2016-06-08

**Authors:** Zi-Zhen Zhang, Gang Zhao, Chun Zhuang, Yan-Ying Shen, Wen-Yi Zhao, Jia Xu, Ming Wang, Chao-Jie Wang, Lin Tu, Hui Cao, Zhi-Gang Zhang

**Affiliations:** 1 Department of General Surgery, Ren Ji Hospital, School of Medicine, Shanghai Jiao Tong University, Shanghai, 200127, P.R. China; 2 Department of Pathology, Ren Ji Hospital, School of Medicine, Shanghai Jiao Tong University, Shanghai, 200127, P.R. China; 3 State Key Laboratory of Oncogenes and Related Genes, Shanghai Cancer Institute, Ren Ji Hospital, School of Medicine, Shanghai Jiao Tong University, Shanghai, 200240, P.R. China

## Abstract

To discover new biomarkers for gastric cancer (GC) diagnose and treatment, we screened the lncRNAs in GC tissues from 5 patients. We found 6 lncRNAs had altered expression, and in the same time, the levels of their neighboring genes (located near 300 kb upstream or downstream of lncRNA locus) were significantly changed. After confirming the results of microarray by qRT-PCR in 82 GC patients, the biological function of LINC00628 was examined through cell proliferation and apoptosis, cell migration and invasion, colony formation assay and cell cycle detection. We confirmed that LINC00628 functions as a GC suppressor through suppressing proliferation, migration and colony formation of cancer cells. Furthermore, LINC00628 can also suppress the tumor size in mouse xenograft models. Although LINC00628 can modulate LRRN2 expression, the GC suppressor function of LINC00628 is not LRRN2 dependent. The result of mRNA microarray indicated that LINC00628 perform GC inhibitor function through long-range modulating cell cycle related genes. Importantly, we confirmed that LINC00628 mainly located in the nucleus and interacted with EZH2, and modulated genes expression by regulating H3K27me3 level. This research shed light on the role of dysregulated LINC00628 during GC process and may serve as a potential target for therapeutic intervention.

Gastric cancer (GC) is the fourth most common cancer and the second most common cause of cancer related death worldwide^1^,^2^. Based on the GLOBOCAN data, a total of 989,600 new stomach cancer cases and 738,000 deaths are estimated to have occurred in 2008, accounting for 8% of the total cases and 10% of total deaths^3^. Although the incidence and mortality of gastric cancer have decreased considerably over the past 50 years, many patients are diagnosed with advanced gastric cancer and have a poor prognosis. Thus, more sensitive GC markers for improving screening, diagnosis, prognostic evaluation and tumor grading is urgently needed.

Depend on the recent discovery, nearly 75% of the human genome is transcribed to RNA, with only about 1.2% being responsible for protein coding, which indicates that a large group of RNA regulators is dedicated to regulating a relatively small amount of effectors^4^,^5^,^6^. Among the newly discovered RNA elements, long non-coding RNAs (lncRNAs) have been identified to function as key regulators of diverse cellular processes, such as development, differentiation, and cell fate as well as disease pathogenesis^7^,^8^. lncRNAs can serve as signal mediators, molecular decoys and scaffold or enhancers of transcription and what is intriguing is that a considerable number of lncRNAs regulate their neighboring gene expression^9^,^10^,^11^.

In this study, to investigate the functions of dysregulated lncRNAs during gastric cancer pathogenesis, five paired cancer- control tissue samples were collected from gastric cancer patients. Microarray analysis was employed to analyze the differentially expressed lncRNAs and their neighboring genes, and subsequently, the results were confirmed by qRT-PCR in the tissue sample of other 82 gastric cancer patients. The correlations between LINC00628 and clinical data were also analyzed. To elucidate the biological function of LINC00628, LINC00628 was overexpressed or knockdown in different gastric cancer cell lines, and the effects on cell proliferation, apoptosis, migration, invasion, cell cycle and nude mouse tumorigenicity were detected. Finally, the effect of overexpressed LINC00628 on protein coding genes were estimated by mRNA microarray.

## Results

### The expression of lncRNAs were disturbed in GC samples

**Figure 1:**
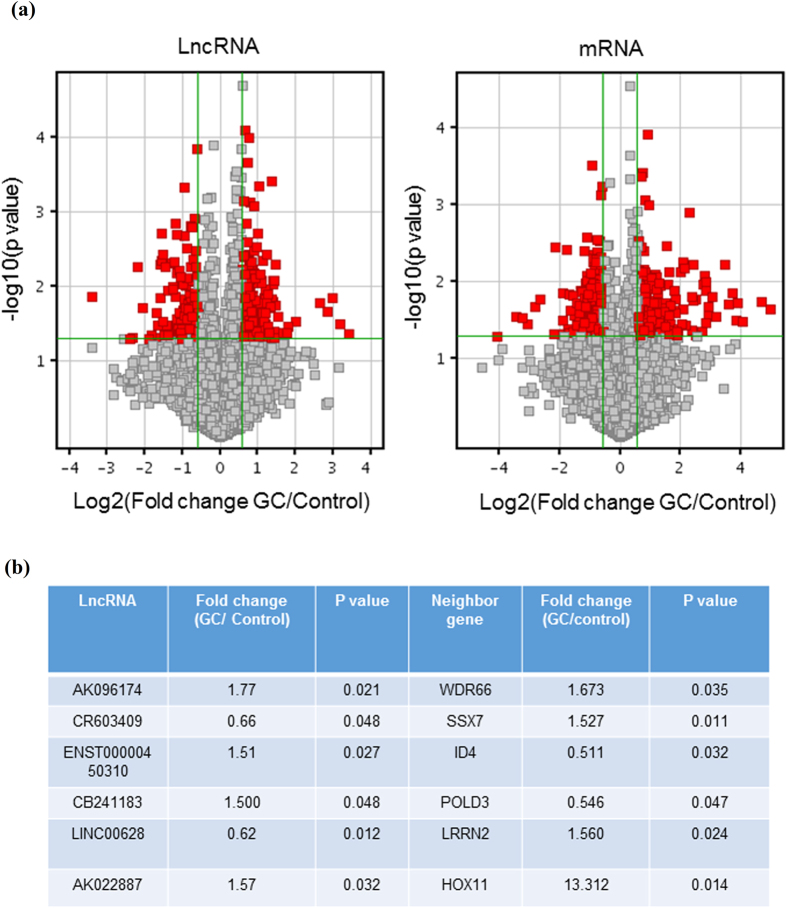
Six lncRNAs and their neighboring genes have disturbed expressions in GC samples and adjacent normal gastric mucosa. Five paired GC tumor tissues and adjacent non-tumor samples were collected. Total RNA was extracted and the expression of 33045 LncRNAs and 30215 mRNAs were determined by microarray. Analysis combining the data of lncRNAs and mRNAs, 6 lncRNAs have significantly altered expression, and in the same time, their neighboring genes (located near 300 kb upstream or downstream of lncRNA locus) level changed significantly. The vertical lines correspond to 1.5-fold up and down and the horizontal line represents a P-value of 0.05. The red point in the plot represents the differentially expressed LncRNAs with statistical significance.

Long non-coding RNA represents a new group of regulators that modulate genes expression transcriptionally or post-transcriptionally. To explore the function of dysregulated lncRNAs during GC pathogenesis, we first detected the expression of lncRNAs in paired GC tissues and adjacent non-tumor tissue from 5 GC patients by microarray assay. Because a considerable number of lncRNAs can function as cis- or trans- regulator for their neighboring genes, so the level of 30215 mRNA was also detected. We found that 220 lncRNAs and 157 mRNAs have a significant higher (>1.5 fold, p < 0.05) expression in GC tissues, meanwhile, 159 lncRNAs and 122 mRNAs are expressed lower significantly (>1.5fold, p < 0.05) in the GC tissues (Fig. 1). Analysis combining the data of lncRNAs and mRNAs, we found 6 lncRNAs have significantly altered expression, and in the same time, their neighboring genes (located near 300 kb upstream or downstream of lncRNA locus) level changed significantly (Fig. 1b).

**Figure 2:**
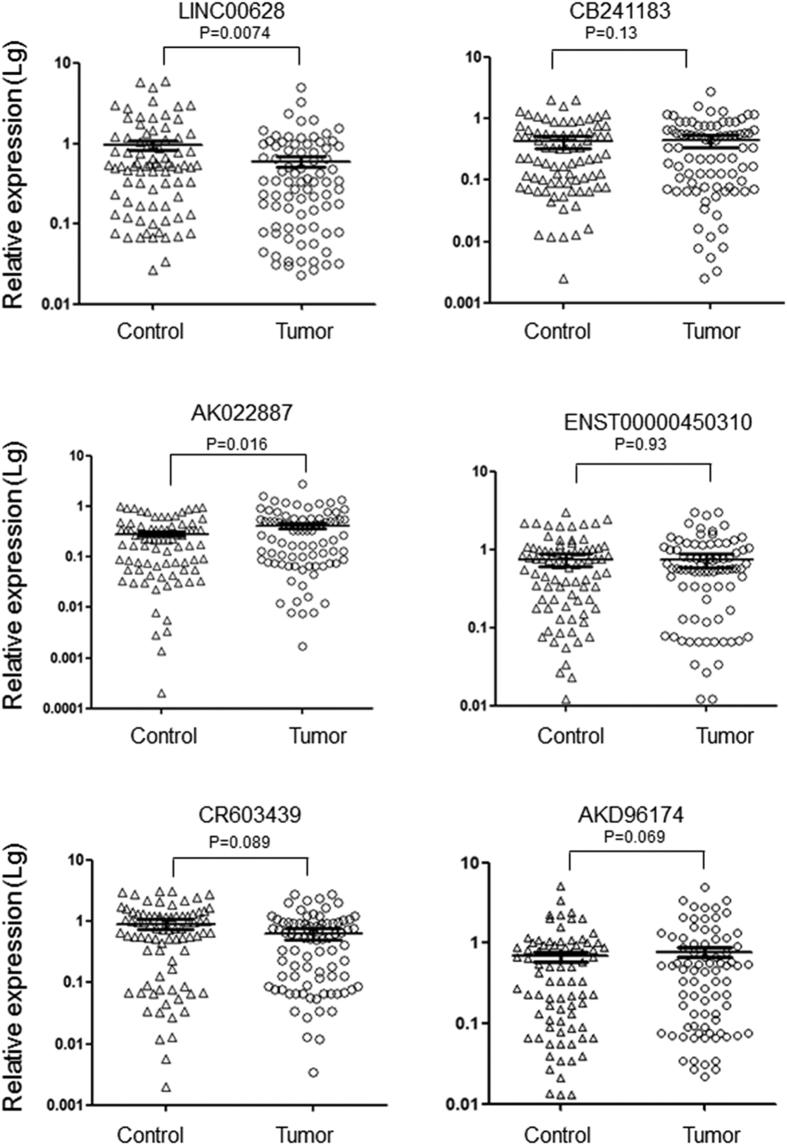
Confirm the expression of six dysregulated lncRNAs in 82 paired GC and normal gastric mucosa samples. The expression of six lncRNAs were detected by qRT-PCR in a sample group consisted by 82 paired GC tumor and adjacent non-tumor tissues. The results were analyzed by student’s t-test and p < 0.05 was considered statistically significant.

**Figure 3:**
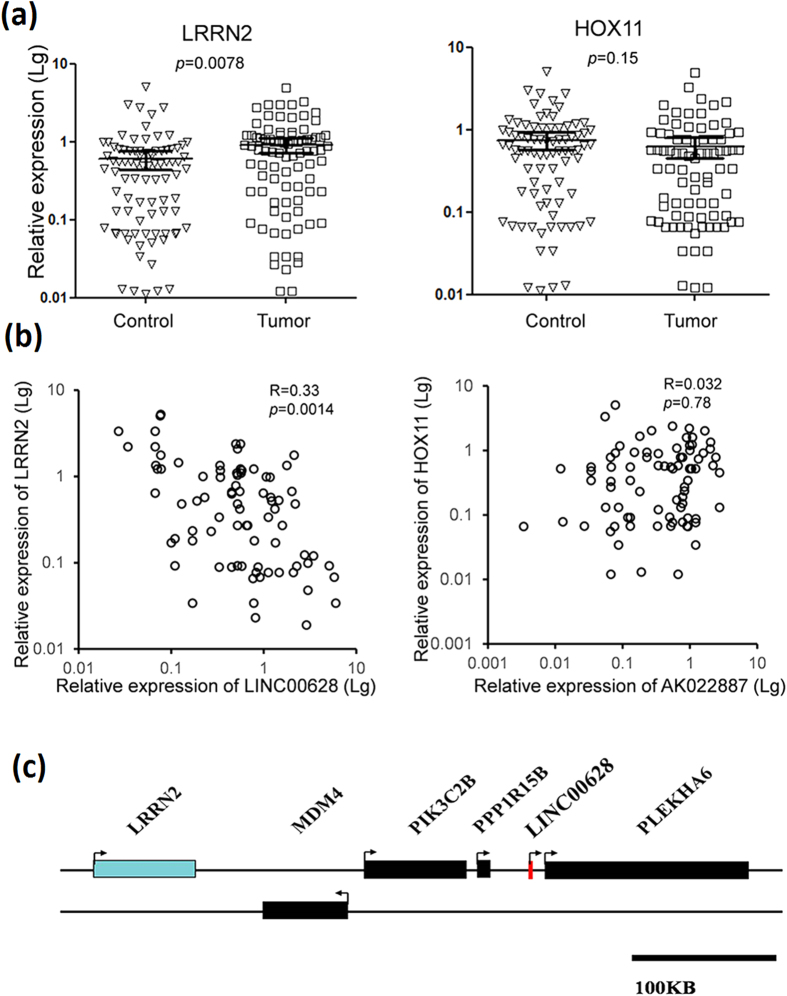
The expression of LRRN2 is negatively correlated with LINC00628 level in GC samples. (**a**) The mRNA levels of LRRN2 and HOX11 were determined by qRT-PCR. The results were analyzed by student’s t-test and p < 0.05 was considered statistically significant. (**b**) χ2-analysises were used to determine the correlation between lncRNAs and their neighboring genes. (**c**) Schematic diagram for exhibiting the relative position between LINC00628 and LRRN2.

Subsequently, the expression of these 6 selected lncRNAs and relevant neighboring genes were detected by qRT-PCR in the GC and adjacent non-tumor samples of 82 GC patients. As exhibited in Fig. 2, LINC00628 down-regulated in the tumor tissues. Meanwhile the expression of leucine-rich repeat neuronal protein 2 (LRRN2) is significantly up-regulated in the tumor tissues (Fig. 3a). The correlation analysis indicates that a positive correlation existed between the expressions of LINC00628 and LRRN2, but no significant relationship is found between AK022887 and LINC00628 (Fig. 3b).

### Reduced LINC00628 level relates to lower survival rate

**Figure 4:**
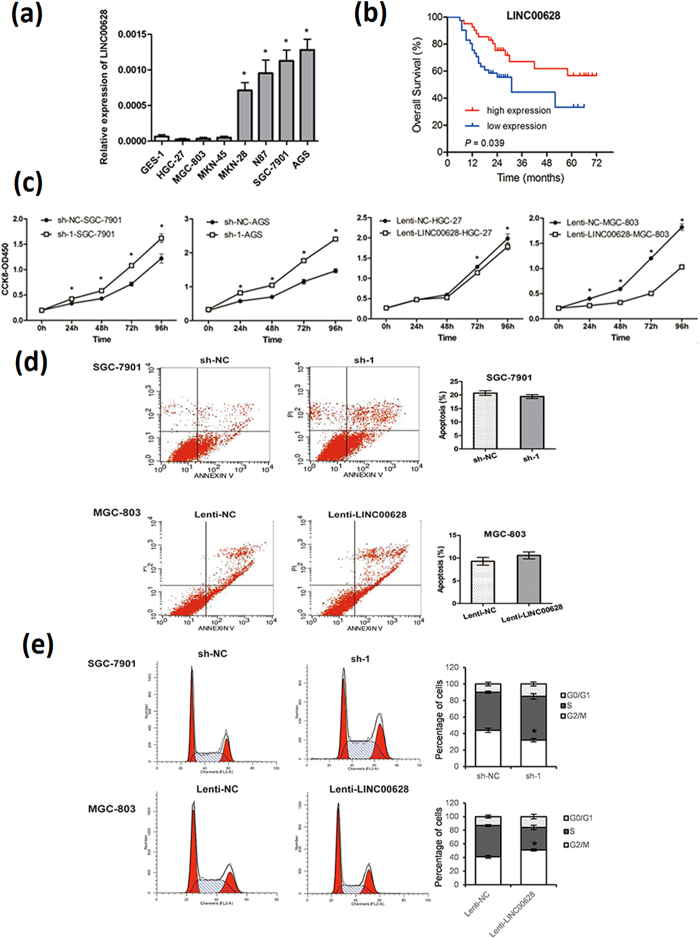
LINC00628 overexpression suppresses GC cells proliferation and causes G0/G1 arrest. (**a**) The expression of LINC00628 in 7 gastric cancer cell lines and normal gastric mucosa cells (GES-1) were detected by qRT-PCR. (**b**) The overall survival curves are presented according to the expression level of LINC00628 in GC patients. (**c**) The function of LINC00628 on cell proliferation was determined by CCK8 assay. (**d**) The numbers of apoptotic cells in LINC00628 knocked down SGC7901 cells and LINC00628 overexpressed MGC803 cells were counted by flowcytometry after annexin V-FITC staining. (**e**) The function ofLINC00628 on cell cycle is determined by flowcytometry after PI staining. *P < 0.05, **P < 0.01.

To further investigate the function of LINC00628, we first detected the expression of LINC00628 expression in different GC and normal gastric mucosa cells. As shown in Fig. 4a, very low level of LINC00628 was found in GES-1, HGC-27, MGC-803 and MKN-45 cells. Oppositely, MKN-28, N87, SGC7901 and AGS cells express more LINC00628 significantly.

According to the relative expression of LINC00628 in the tissue samples, 82 GC patients were divided into two groups and survival analysis were used to examine the function of LINC00628 on the GC patients’ survival time. As shown in Fig. 4b, the patients with higher LINC00628 level in the GC tissues have a higher survival rate, suggesting that LINC00628 may function as a GC suppressor.

### LINC00628 represses GC development through inhibiting cells proliferation, migration and invasion

**Figure 5:**
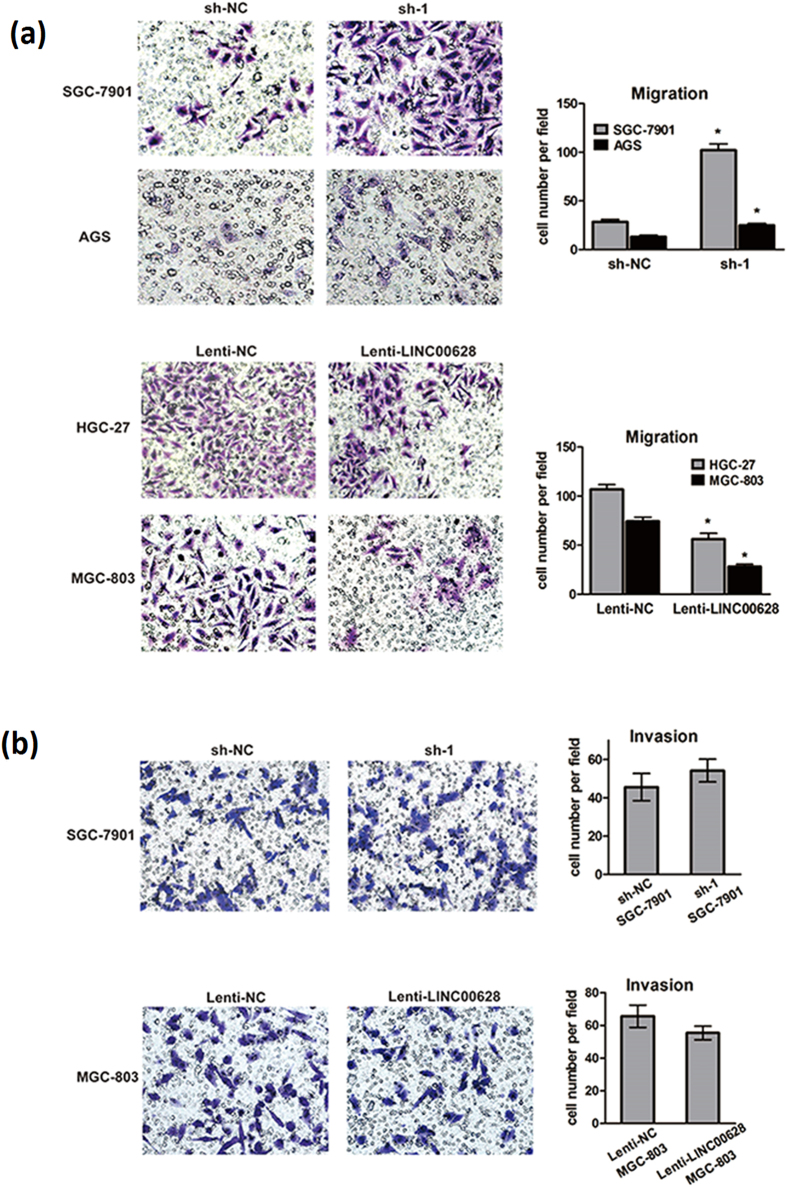
LINC00628 overexpression suppresses GC cells migration and invasion. A typical Transwell assay was used to examine the impact of LINC00628 on GC cells migration and invasion. 3 × 10^4^ cells in 200 μL serum-free medium were seeded to the top chamber and 500 μL medium with high concentration of serum was added to the bottom. After 12 h, Filters were then submerged in 4% PFA for 15 min and cells on the upper surface were removed by cotton swabs. The cells on the lower surface were stained with hematoxylin-eosin. Ten random fields were selected to determine the average number of cells per view field. For cell invasion assays, the procedure was similar to the cell migration assay, except transwell membranes were precoated with 24 μg/μl Matrigel (BD bioscience, Franklin Lakes, NJ, USA) and the cells were incubated for 8 hr at 37 °C in a 5% CO_2_ atmosphere. *P < 0.05.

To further confirm the GC suppressor function of LINC00628, we detected the cell proliferation, apoptosis, migration and invasion capacity and cell cycle variation in GC cells with overexpressed or down-regulated LINC00628. As shown in Fig. 4c, the cell proliferation was significantly up-regulated when LINC-00628 was knocked down in SGC-7901 and AGS cells. Oppositely, the cell proliferation was down-regulated in HGC-27 and MGC-803 cells in which LINC00628 was overexpressed. However, the cell apoptosis is not significantly changed when LINC00628 was up-regulated in MGC-803 cells or knocked-down in SGC7901 cells (Fig. 4d). But what is intriguing is that when LINC00628 was up-regulated, a significant G0/G1 arrest was observed, and vice versa (Fig. 4e). Furthermore, the migration capacity of GC cells was up-regulated when LINC00628 was knocked down, and down-regulated when LINC00628 was overexpressed (Fig. 5). These data indicated that LINC00628 functions as a GC suppressor by suppressing proliferation and migration of GC cells.

**Figure 6:**
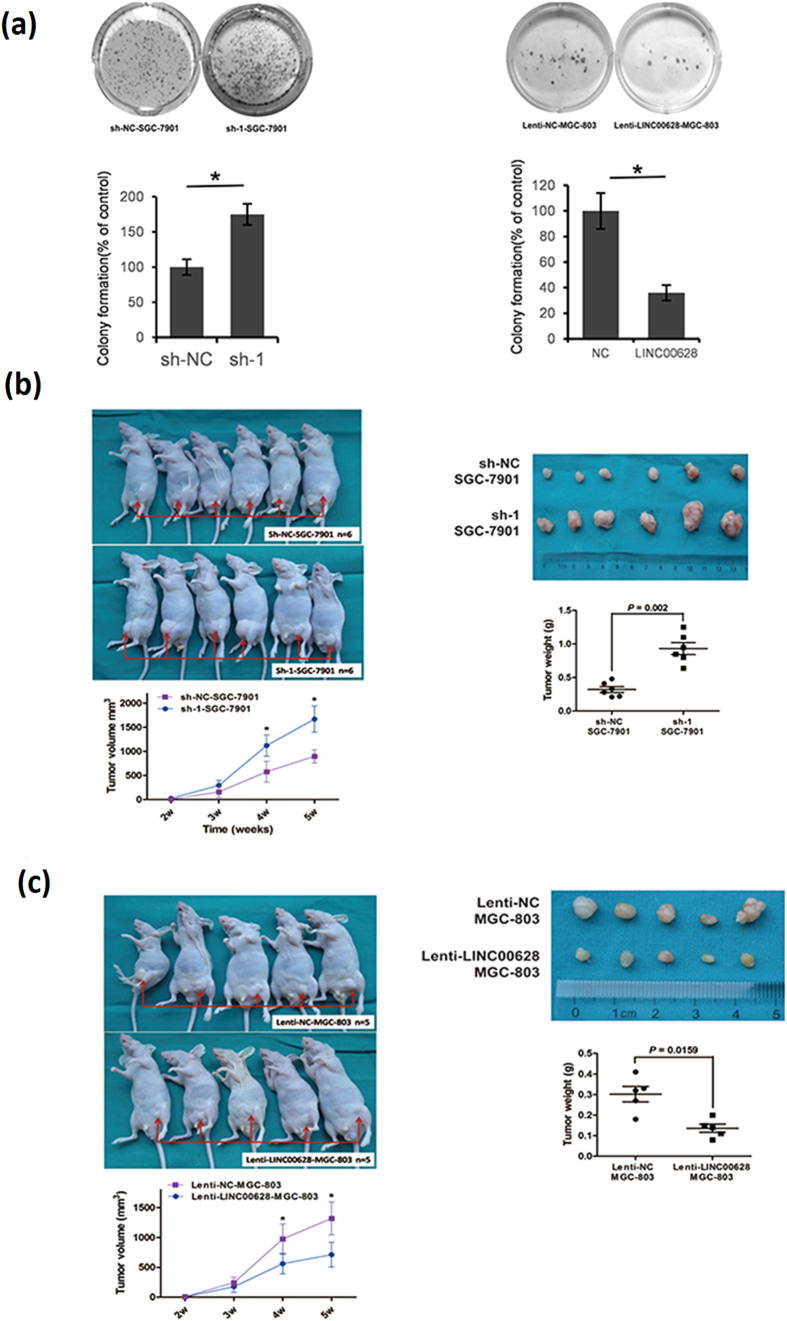
LINC00628 function as a GC suppressor via suppressing GC tumorigenicity. (**a**) LINC00628 knocked down SGC-7901 cells and LINC00628 overexpressed MGC-803 cells were separately plated in 60 mm plates at a density of 1000 cells per plate. For each clone, three independent wells were examined. After 2 weeks of incubation at 37 °C and 5% CO_2_, colonies were stained with 0.2% crystal violet and counted. (**b,c**) Stable SGC-7901 cell with deleted LINC00628 and MGC-803 cell with overexpressed LINC00628 were injected subcutaneously into both hind limbs of the nude mice for 5 weeks. The tumor size was detected every week from the second week from injection. The tumor weight was measured post sacrifice. Results were analyzed by student’s t-test and p < 0.05 was considered significant. *p < 0.05.

To further identify the function of LINC00628 on tumorigenicity, we did the colony formation assay. As shown in Fig. 6a, the colonies formed by SGC-7901 were fewer and smaller when LINC00628 was knocked down. Meanwhile, LINC-00628 overexpressed MGC-803 cells formed more and larger colonies. Subsequently, we confirmed the function of LINC00628 *in vivo* by injecting SGC-7901 sh-NC or SGC-7901 sh-1 or MGC-803 lenti-NC or MGC-803 lenti-LINC-00628 stable transfected cells into both hind limbs of the nude mice for 5 weeks. Tumor volume was evaluated over time up to 5 weeks and tumor weight was detected at the fifth week. As shown in Fig. 6b, reduced LINC00628 expression is associated with increased tumor burden. Meanwhile, up-regulated LINC00628 contributes to decreased tumor burden (Fig. 6c).

### LINC00628 functions as a GC suppressor not through modulating the expression of LRRN2

**Figure 7:**
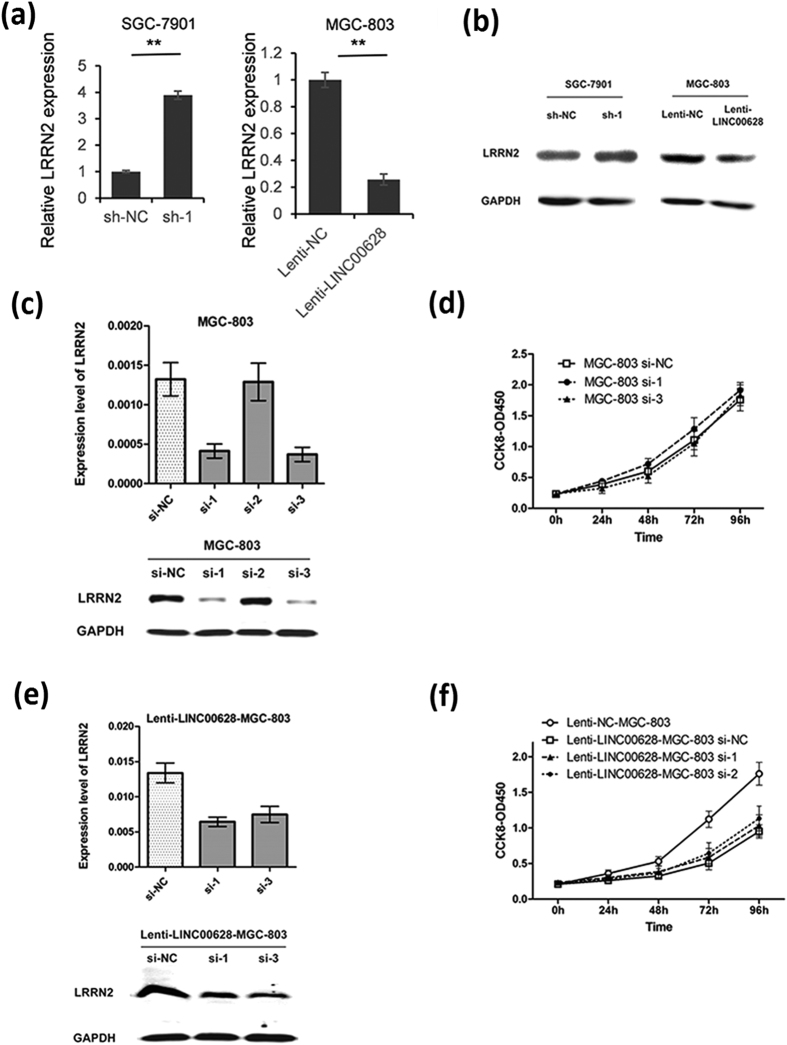
LINC00628 negatively regulated LRRN2 expression but the LRRN2 cannot modulate cell proliferation in GC cells. The expression of LRRN2 in LINC00628 knocked down SGC-7901 or overexpressed MGC-803 cells were detected by qRT-PCR (**a**) and western blot (**b**). SiRNAs for LRRN2 were transfected into MGC-803 cells. 48 h after transfection, CCK8 assay was processed to determine the cell viability (**d**). The mRNA and protein level of LRRN2 was detected by qRT-PCR and western blot (**c**). SiRNAs for LRRN2 were transfected into Lenti-LINC00628-MGC-803 cells. 48 h after transfection, CCK8 assay was processed to determine the cell viability (**f** ). The mRNA and protein level of LRRN2 was detected by qRT-PCR and western blot (**e**).

To further explore the mechanism of how LINC00628 plays as a GC suppressor, we first examined that whether the function of LINC00628 is related to modulating the expression of LRRN2. As shown in Fig. 7a,b, the mRNA and protein level of LRRN2 was up-regulated significantly in SGC-7901 cells when LINC00628 was knocked down. Meanwhile, LRRN2 expression was down-regulated in MGC-803 cells when LINC00628 was overexpressed. However, the cell proliferation capacity was not changed in the LRRN2 knock-down MGC-803 and LINC00628 overexpressed MGC-803 cells. These results indicated that LINC00628 suppresses GC cell proliferation through LRRN2 independent manner.

**Figure 8:**
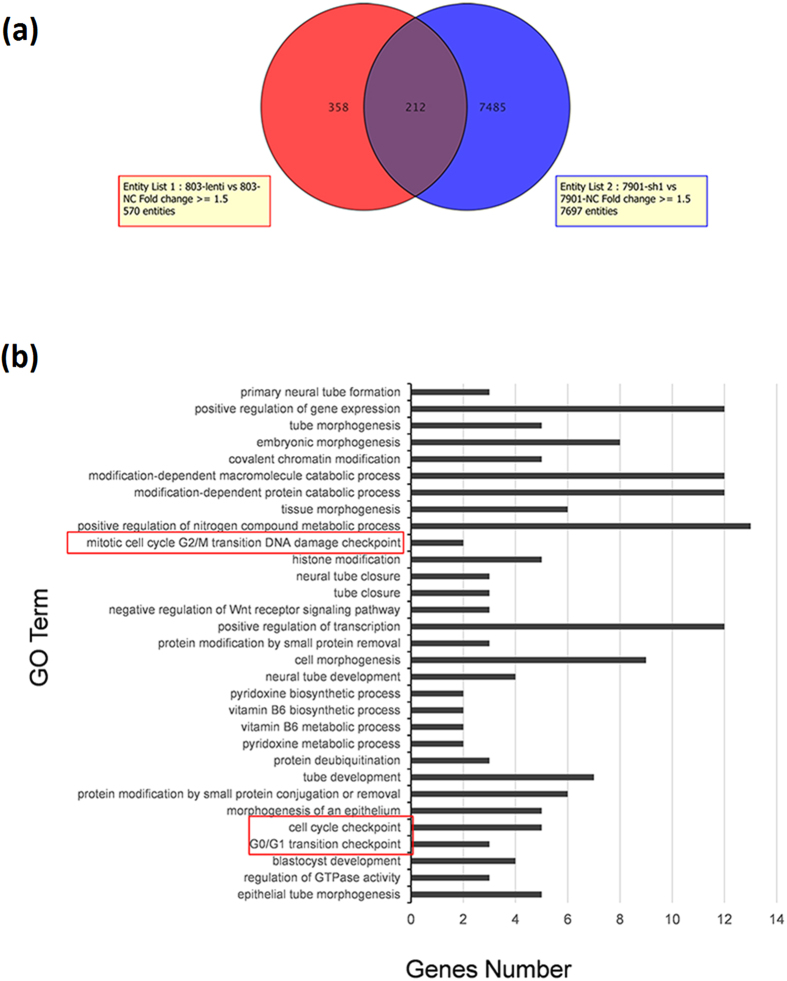
LINC00628 function as a GC suppressor via modulating cell cycle controlling genes by long range method. (**a**) Schematic diagram of the strategy for analyzing LINC00628 regulated genes. Microarray analysis was employed to determine mRNA alteration in the LINC00628 knocked down SGC-7901 and overexpressed MGC-803 cells. (**b**) GO analysis of the genes regulated by LINC00628.

Through analyzing the data of mRNA profile of LINC00628 overexpressed MGC-803 cells and LINC00628 knocked down SGC-7901 cells, we find a clue to annotate the biological function of LINC00628. As shown in Fig. 8a, there are 212 genes significantly changed in LINC00628 overexpressed MGC-803 and LINC00628 knocked down SGC7901 cells. Results of GO analysis indicated that 5 out of 212 genes are related to cell cycle modulation.

**Figure 9:**
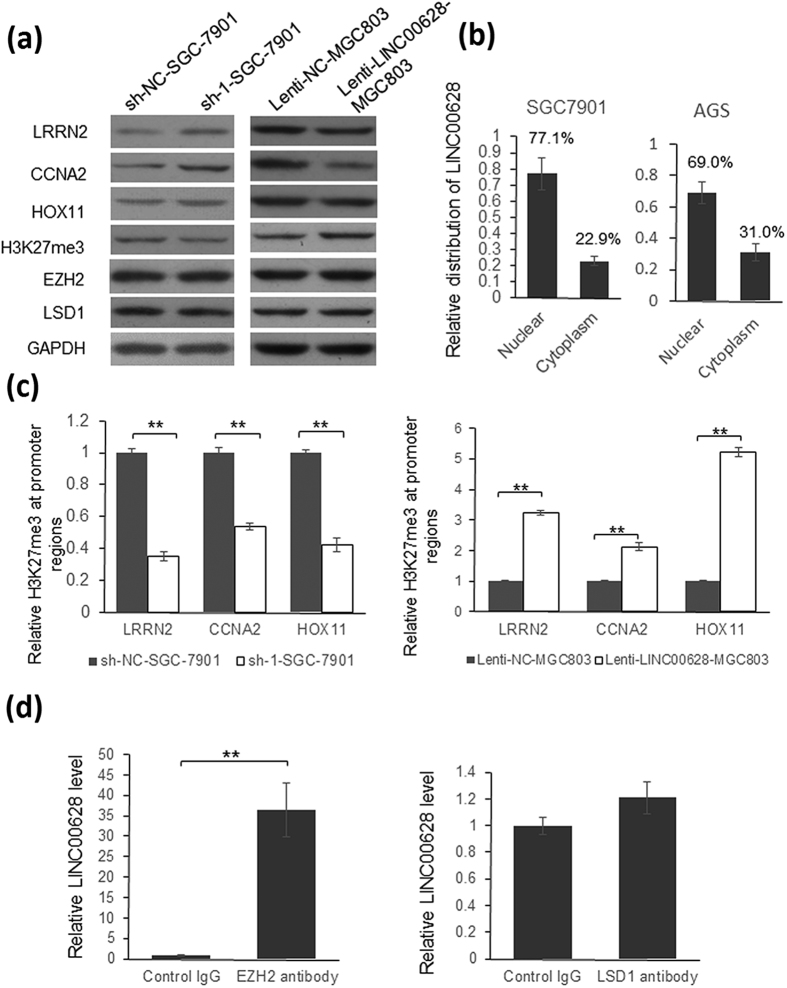
LINC00628 regulate genes expression by modulating H3K27m3 level. (**a**) Immunoblotting was employed to detect the expression of LRRN2, CCNA2, HOX11, H3K27me3, EZH2 and LSD1 in LINC00628 knock down SGC-7901 and LINC00628 overexpressed MGC803 cells. The signal of GAPDH was used as loading control. (**b**) RNA was extracted from nucleus and cytoplasm separately and the expression of LINC00628 was detected by qRT-PCR. (**c**) Chromatin immunoprecipitation was processed using anti-H3K27me3 antibody in LINC00628 knock down SGC-7901 and LINC00628 overexpressed MGC803 cells. The promoter regions of LRRN2, CCNA2 and HOX11 were detected by qRT-PCR. (**d**) RNP immunoprecipitation was processed using anti-EZH2 or anti-LSD1 antibody, and RNA was extracted from the pellets. The relative level of LINC00628 was detected by RT-qPCR. The results were analyzed by student’s t-test and p < 0.05 was considered statistically significant. **p < 0.01.

There are reports indicate that some lncRNAs modulate genes expression by changing histone methylation like HORAIR(12), TUG1(13), Linc-MAF-4(14) and *et al.*, so we hypothesized that the function of LINC00628 may be related to histone methylation. The expression of H3K27me3, LSD1 and EZH2 was detected by immunoblotting in SGC-7901 and MGC803 cells. As shown in Fig. 9a, H3K27me3 level was reduced in LINC00628 knock down SGC-7901 cells and up-regulated in LINC00628 overexpressed MGC803 cells. Meanwhile, the expression of EZH2 and LSD1 was not significantly changed, and the most part of LINC00628 was detected in the cell nucleus, suggesting that LINC00628 may regulate histone methylation by interacting with histone methylation related proteins (Fig. 9b). To confirm this hypothesis, we did ChIP assay using anti-H3K27me3 antibody, and detected the sequence of promoter region of LRRN2, CCNA2 and HOX11 by qRT-PCR. As shown in Fig. 9c, the H3K27me3 level in the promoter region of LRRN2, CCNA2 and HOX11 was down-regulated significantly in LINC00628 knockdown SGC7901 cells and significantly up-regulated in LINC00628 overexpressed MGC803 cells. Subsequently, RNP co-IP and qRT-PCR were processed using EZH2 or LSD1 antibodies. As shown in Fig. 9d, LINC00628 can be enriched by EZH2 antibody not by LSD1 antibody, indicating a direct interaction between LINC00628 and polycomb repressive complex 2.

## Discussion

Recently, several lncRNAs have been found to be related to gastric cancer pathogenesis. HOTAIR, function as a scaffold of histone modification complexes and promoter of breast cancer metastasis, was reported to have nearly the same function in the gastric adenocarcinoma^12^,^13^,^14^. Overexpressed GAIPLNC, a 924 bp lncRNA, was found to be associated with CD44-dependent cell invasiveness and associates with poor prognosis of patients with gastric cancer^15^. Long noncoding RNA MRUL was also reported to promote ABCB1 expression in multidrug-resistant gastric cancer cell sublines^16^.

To discover new biomarkers and candidate compounds for GC diagnose and treatment, we first screened the lncRNAs in GC tumor and adjacent control samples from 5 patients. We found 6 lncRNAs have significantly altered expression, and in the same time, the level of their neighboring genes (located near 300 kb upstream or downstream of lncRNA locus) was changed significantly. After confirming the results of microarray, the biological function of LINC00628 was examined through cell proliferation and apoptosis assays, cell migration and invasion assays, colony formation assay and cell cycle detection. We confirmed that LINC00628 function as a GC suppressor especially via suppressing proliferation, migration and colony formation capacities of GC cells. Furthermore, LINC00628 can also suppress the tumor size in mouse xenograft models. Although LINC00628 can modulate LRRN2 expression, the GC suppressor function of LINC00628 is not LRRN2 dependent. The result of mRNA microarray indicated that LINC00628 perform the GC inhibitor function through long-range modulating cell cycle related genes.

LINC00628 located in the 2^nd^ intron of PLEKHA6 in chromosome 1q32.1, and the mature RNA is 1290 bp and poly A tailed. The function of LINC00628 has not been reported yet. LRRN2 was located at about 30 kb loci up-stream of LINC00628. LRRN2 was first reported to have an up-regulated expression in glioma samples and its function may related to cell-adhesion and signal transduction^17^,^18^. Our research indicated that the expression of LRRN2 has a negative correlation with the expression of LINC00628 in clinical samples and LRRN2 was regulated by LINC00628 in GC cells. However, the cell proliferation capacity was not changed in the LRRN2 knock-down GC cells. Since the expression of LRRN2 is really up-regulated in GC samples, the function of LRRN2 in GC needs to be further examined.

In the present study, we have provided a proof of principle study of searching a biomarker potential lncRNA using lncRNAs expression profiles. Most of previous studies have explored the feasibility of using mRNA profiles for predicting cancer or discriminating between cancer subtypes but lncRNA based predictive biomarkers are rarely reported. Owing to new RNA detection technology, we have the chance to study GC related lncRNAs. Here, we presented that lower LINC00628 expression is related to lower survival rate of GC patients which suggesting that LINC00628 may be used as prediction markers for prognostic.

LncRNAs are a group of versatile physiological mediators and a part of them have been shown to regulate transcription of neighbouring genes on the same chromosome mainly through cis mechanisms^19^,^20^. Meanwhile, lncRNA transcripts have also been confirmed to regulate gene expression in trans, without influencing transcription of their genomically neighbouring genes^21^,^22^. In this study, our results demonstrated functions of LINC00628 in controlling GC cells proliferation and migration. In addition to conveying these functions, via LINC00628 overexpression and knockdown, we discovered a group of distal genes the expression of which are regulated by LINC00628. Dysregulation of these genes may contributes to the GC pathogenesis although LRRN2, a local gene which is regulated by LINC00628, the disturbed expression of which was also found related to GC. Since lncRNAs can regulate distal genes expression through many different ways, such as modulating histone modification and absorbing miRNAs. Further research should be focused on studying those proteins which LINC00628 interacts with.

To understand the mechanism of how LINC00628 modulate the expression of target genes, we detected the level of H3K27me3, EZH2 and LSD1. The expression of H3K27me3 has a negative correlation with LINC00628, however, the expression of EZH2 and LSD1 is not significantly changed by LINC00628. After ChIP assay using anti-H3K27me3 antibody, we found that the H3K27me3 level in the promoter region of LRRN2, CCNA2 and HOX11 was down-regulated significantly in LINC00628 knockdown SGC7901 cells and significantly up-regulated in LINC00628 overexpressed MGC803 cells. Since CCNA2 and HOX11 are both oncogenes and ultra-methylation of H3K27 at their promoter regions may directly lead to the reduction of their mRNA level. So these results may explain why LINC00628 functions as a tumor suppressor. Furthermore, we confirmed a direct interaction between LINC00628 and EZH2, suggesting that LINC00628 may regulate H3K27me3 level by guiding PRC2 to target genes selectively. However, genes expression can be modulated by multiple ways, full scale function of LINC00628 needs to be further unveiled.

In conclusion, we have documented that LINC-00628 has a lower expression GC tissues than adjacent healthy controls, and functions as a GC suppressor through repressing GC cell proliferation, migration and tumorigenicity. Overexpression of LINC00628 can also induce G0/G1 arrest in GC cells. LINC00628 negative regulates its neighboring gene - LRRN2 expression, but the GC suppressor function of LINC00628 mainly depends on modulating cell cycle.

## Materials and Methods

### Tissue collection

The tissue samples used for microarray were collected from 5 patients in 2009 between October and December. The tumorous tissues were taken at from the center of the tumor and non-tumorous samples were taken at a distance of at least 5 cm from the tumor, and all tissues were examined histologically.

82 other gastric cancer samples were obtained from patients who had underwent surgery at Renji Hospital of Shanghai Jiaotong University School of Medicine between 2007 January and 2012 September, and were diagnosed with gastric cancer (stages I to IV, according to the seventh edition of the AJCC Cancer Staging Manual) based on histopathological evaluation. No local or systemic treatment was conducted in these patients before the operation. All specimens were immediately frozen in liquid nitrogen, and stored at −80 °C until RNA extraction. The study was approved by the Research Ethics Committee of RenJi Hospital, School of Medicine, Shanghai Jiao Tong University. Informed consents were obtained from all patients. All of the procedures were done in accordance with the approved guidelines,

We divided the 82 patients into two groups according to the ratio of their cancer/normal tissue expression levels of LINC00628, as ≥1.0 or <1.0 for the cancer/noncancerous tissues expression levels of LINC00628. There were 39 cases (48.8%) in the high LINC00628 group and 43 cases (51.2%) in the low LINC00628 expression group.

### Cell line and cell culture

Human gastric cancer cell lines SGC7901, AGS, HGC-27 and MGC-803 were obtained from China Infrastructure of Cell Line Resources (5 Dong Dan San Tiao, Beijing, 100005, China) and cultured in RPMI 1640 Medium containing 10% fetal bovine serum (Hyclone, Logan, UT, USA), 100 IU/ml penicillin and 10 mg/mL streptomycin. All cells were maintained at 37 °C under an atmosphere of 5% CO_2_.

### RNA purification, cDNA synthesis and qRT-PCR

Total RNA was extracted from tissues by using Trizol (Invitrogen, Carlsbad, CA, USA) according to the manufacturer’s instructions. cDNA synthesis was processed by using One-Step gDNA Synthesis SuperMix (Beijing TransGen Biotech Co., Ltd, Beijing, China). qRT-PCR was performed using the Sybr green reaction mix and ABI 7500 Real Time PCR System (Applied Biosystems, Foster City, CA, USA). For all of the qRT-PCRs, glyceraldehyde-3-phosphate dehydrogenase (GAPDH) was measured as an internal control.

### Poly(A)+ and poly(A)− RNA separation

Total RNAs were prepared using Trizol Reagent (Life Technologies, Carlsbad, CA, USA). After treatment with DNase I (DNA-free kit; Ambion, Austin, TX, USA), total RNAs were incubated with oligo(dT) magnetic beads to isolate either poly(A)+ RNAs, which were bound to beads, or poly(A)− RNAs, which were present in the flowthrough after incubation. Oligo(dT) magnetic bead selection was performed three times to ensure pure poly(A)+ or poly(A)− populations.

### Microarray expression assays

The RNA samples were used to perform the microarray hybridization by using the Human LncRNA Array v2.0 (8 × 60 K, Arraystar, Rockville, MD, USA) and the bioinformatic analysis was performed by Kang Chen Bio-tech. The microarray data were submitted to Gene Expression Omnibus (GEO) Database (http://www.ncbi.nlm.nih.gov/geo/) and accession number is GSE51308.

### Lentiviral infection

Lentivirus was produced by the co-transfection of 293FT cells with a pLenti vector (GLV2-shControl or GLV2-shLINC00628 or GLV2-LINC00628 or GLV2-NC) and lentiviral packaging vectors (pPACK-GAG, pPACK-REV, pPACK-VSV-Gp). Lentivirus- containing supernatant was harvested 48 h post-transfection, purified by centrifugation and stored at −80 °C. BGC-823 and SGC7901 cells were infected with viral supernatant containing polybrene. 24 h post-infection, stable GC cells with depleted or overexpressed LINC00628 were selected by culturing in puromycin.

### Cell viability and proliferation assays

Colony formation assays were used to assess the survival capacity of SGC7901 and BGC-823 cells with and without LINC00628. Five hundred cells were seeded into 6 cm dishes. After 14 days of culture, the colonies that formed were fixed with methanol and were then stained with 0.5% crystal violet and manually counted.

Samples of 2 × 10^3^ cells/well for SGC7901 and BGC823 were plated into 96-well plates in triplicate and were allowed to adhere overnight. Cells proliferation abilities were determined by CCK8 (Dojindo Laboratories, Shanghai, China).

### Cell cycle assay and apoptosis assay

Cells were harvested after lentivirus infection for 48 h, washed twice with cold PBS and fixed in 70% alcohol at −20 °C overnight. After fixation, cells were stained with PI (propidium iodide, Sigma) at a final concentration of 50 ng/mL in the dark tubes at 25 °C for 30 min to detect cell cycle and stained with 5 μL of annexin V-FITC to detect apoptosis. The stained cells were then analyzed by using flowcytometry BD FACS Canto II (BD, USA). The flowcytometry assay was repeated three times.

### Cell migration and invasion assay

A typical transwell assay (Costar, 6.5 mm diameter, 8 μm pore size, with or without a matrigel treating) was used. 3 × 10^4^ cells in 200 μL serum-free medium were seeded to the top chamber and 500 μL medium with high concentration of serum was added to the bottom. After 12 h, Filters were then submerged in 4% PFA for 15 min and cells on the upper surface were removed by cotton swabs. The cells on the lower surface were stained with hematoxylin-eosin. Ten random fields were selected to determine the average number of cells per view field. For cell invasion assays, the procedure was similar to the cell migration assay, except transwell membranes were precoated with 24 μg/μl Matrigel (BD bioscience, Franklin Lakes, NJ, USA) and the cells were incubated for 8 hr at 37 °C in a 5% CO_2_ atmosphere.

### Colony formation assay

LINC00628 knocked down SGC-7901 cells and LINC00628 overexpressed MGC-803 cells Parental were separately plated in 60 mm plates at a density of 1000 cells per plate. For each clone, three independent wells were examined. After 2 weeks of incubation at 37  °C and 5% CO_2_, colonies were stained with 0.2% crystal violet and counted.

### Western blotting

Equal amounts of total protein were separated by SDS-PAGE and transferred to nitrocellulose membranes (Amersham Pharmacia Biotech, St. Albans, Herts, UK) using an electrotransfer unit. The membranes were blocked with 5% nonfat dry milk and then incubation with rabbit anti-LRRN2 polyclonal antibody (Abcam, Cambridge, MA, USA, ab125921), or rabbit anit-EZH2 polyclonal antibody (Abcam, Cambridge, MA, USA, ab3748), or rabbit anti-LSD1 polyclonal antibody (Abcam, Cambridge, MA, USA, ab17721), or mouse anti-CCNA2 monoclonal antibody (Abcam, Cambridge, MA, USA, ab38), or rabbit anti-HOX11 polyclonal antibody (Abcam, Cambridge, MA, USA, ab94528), or mouse anti-GAPDH monoclonal antibody(Abcam, Cambridge, MA, USA, ab8245). The specific protein antibody complex was detected by using horseradish peroxidase conjugated goat anti-rabbit or rabbit anti-mouse IgG. Detection by the chemiluminescence reaction was carried using the ECL kit (Pierce, Appleton, WI, USA). The GAPDH signal was used as a loading control.

### *In vivo* tumor studies

Four week old male nude mice were purchase from the Shanghai National Center for Laboratory Animals, Chinese Academy of Science. They were maintained in specific pathogen free facilities. Stable SGC7901 cell with deleted LINC00628 and MGC-803 cell with overexpressed LINC00628 were injected subcutaneously into both hind limbs of the nude mice for 5 weeks. The tumor size was detected every week from the second week from injection. The tumor weight was measured post sacrifice. The methods were carried out in accordance with the approved guidelines. All animal experimental protocols were approved by Animal Center of School of Medicine, Shanghai Jiao Tong University.

### Ribonucleoprotein (RNP) co-immunoprecipitation

Cells were irradiated with 150 mJ/cm2 of UV radiation for crosslinking and then were washed in cold PBS, scraped and then lysed with a buffer containing 0.5% NP40, 0.5 mM DTT, 20 mM Tris-HCL pH 7.5, 150 mM KCL, 2 mM EDTA, 1 mM NaF and inhibitors of RNases, proteases and phosphatases. 10% of total lysate was removed and kept as the input samples and the remainder used for immunoprecipitation. 10 μg of anti-EZH2 or anti-LSP1 or anti-IgG antibodies were bound to sepharose beads in the presence of heparin. Pre-cleared lysates were then incubated with the appropriate antibody-bound beads at 4 °C for 4 hours. After washed for three times, RNA was extracted from the pellets using Trizol reagent.

### Chromatin immunoprecipitation (ChIP)

ChIP was performed in SGC7901 and SGC803 cells. Cells were cross-linked using 1% formaldehyde for 10 min followed by quenching with 0.125 M glycine for 5 min. Cross-linked cells were lysed using lysis buffer (50 mM Hepes pH 7.5, 140 mM NaCl, 1 mM EDTA, 1% triton X-100, 0.1% sodium deoxycholate, 1 tablet of Roche protease inhibitor per 10 mL lysis buffer). The whole cell lysates were sonicated for 15 min. Sonicated lysates were then centrifuged at 2500 g for 10 min, and the supernatant was used to perform ChIP using mouse anti-Histone H3 monoclonal antibody (Abcam, Cambridge, MA, USA, ab6002). For quantification of enrichment of LRRN2, CCNA2 and BRCC3 at promoter regions, qPCR was performed using Sybr green reaction mix and ABI 7500 Real Time PCR System (Applied Biosystems, Foster City, CA, USA).

### Nuclear/Cytoplasmic RNA fractionation from cells

SGC7901 or AGS cells were grown in 10 cm dishes. Cells were collected and centrifuged at 1000 rpm for 5 min and rinsed with ice-cold PBS. Cell pellets were suspended by gentle pipetting in 200 μL lysis buffer A (10 mM Tris (pH8.0), 140 mM NaCl, 1.5 mM MgCl2, 0.1% Igepal, 2 mM vanadyl ribonucleoside complex), and incubated on ice for 5 min. 1/5 of the total RNAs were extracted by Trizol reagent. The rest of the lysate was centrifuged at 1000 g for 3 min at 4 °C to isolate the cytoplasmic fraction and pellet the nuclei. Cytoplasmic fractions were further centrifuged at maximum speed and nuclear pellets underwent two additional washes with 160 μL lysis buffer A before extraction with Trizol.

### Statistical analysis

Data were expressed as mean ± standard deviation (SD). Statistical analysis was performed using one- way ANOVA or a sample independent t test. The correlation analysis were analyzed by χ2-analysis. The survival times of different groups of patients were analyzed using the Kaplan-Meier method. Differences were considered significant at a P value of <0.05.

## Additional Information

**How to cite this article**: Zhang, Z.-Z. *et al.* Long non-coding RNA LINC00628 functions as a gastric cancer suppressor via long-range modulating the expression of cell cycle related genes. *Sci. Rep.* **6**, 27435; doi: 10.1038/srep27435 (2016).
